# Whole exome sequencing and single nucleotide polymorphism array analyses to identify germline alterations in genes associated with testosterone metabolism in a patient with androgen insensitivity syndrome and early-onset colorectal cancer

**DOI:** 10.1186/s40880-016-0115-1

**Published:** 2016-06-07

**Authors:** Vittoria Disciglio, Andrea Devecchi, Orazio Palumbo, Massimo Carella, Donata Penso, Massimo Milione, Giorgio Valle, Marco Alessandro Pierotti, Marco Vitellaro, Lucio Bertario, Silvana Canevari, Stefano Signoroni, Loris De Cecco

**Affiliations:** Department of Experimental Oncology and Molecular Medicine, Functional Genomics and Bioinformatics, Fondazione IRCCS Istituto Nazionale dei Tumori, 20133 Milan, Italy; Medical Genetics Unit, IRCCS Casa Sollievo della Sofferenza, 71013 San Giovanni Rotondo FG, Italy; Department of Pathology and Laboratory Medicine, Fondazione IRCCS Istituto Nazionale Tumori, 20133 Milan, Italy; Department of Biology, University of Padova, 35131 Padua, Italy; Fondazione IRCCS Istituto Nazionale dei Tumori, 20133 Milan, Italy; Hereditary Digestive Tract Tumors Unit, Department of Preventive and Predictive Medicine, Fondazione IRCCS Istituto Nazionale dei Tumori, 20133 Milan, Italy; Colorectal Surgery Unit, Department of Surgery, Fondazione IRCCS Istituto Nazionale dei Tumori, 20133 Milan, Italy

**Keywords:** Androgen insensitivity syndrome, Androgen receptor, Colorectal cancer, Single nucleotide polymorphism array, Testosterone, Whole exome sequencing

## Abstract

**Background:**

Androgen insensitivity syndrome (AIS), a disorder of sexual development in 46, XY individuals, is caused by loss-of-function mutations in the androgen receptor (*AR*) gene. A variety of tumors have been reported in association with AIS, but no cases with colorectal cancer (CRC) have been described.

**Case presentation:**

Here, we present a male patient with AIS who developed multiple early-onset CRCs and his pedigree. His first cousin was diagnosed with AIS and harbored the same *AR* gene mutation, but with no signs of CRC. The difference in clinical management for the two patients was that testosterone treatment was given to the proband for a much longer time compared with the cousin. The CRC family history was negative, and no germline mutations in well-known CRC-related genes were identified. A single nucleotide polymorphism array revealed a microduplication on chromosome 22q11.22 that encompassed a microRNA potentially related to CRC pathogenesis. In the proband, whole exome sequencing identified a polymorphism in an oncogene and 13 rare loss-of-function variants, of which two were in CRC-related genes and four were in genes associated with other human cancers.

**Conclusions:**

By pathway analysis, all inherited germline genetic events were connected in a unique network whose alteration in the proband, together with continuous testosterone stimulation, may have played a role in CRC pathogenesis.

**Electronic supplementary material:**

The online version of this article (doi:10.1186/s40880-016-0115-1) contains supplementary material, which is available to authorized users.

## Background

Tissue resistance to androgens (i.e., testosterone and its 5α-reduced product dihydrotestosterone) leads to androgen insensitivity syndrome (AIS). AIS is the most well-known specific cause of the 46,XY disorder of sex development (DSD), but estimates of its prevalence vary widely, depending on the molecular assessment method used for diagnosis [1–3]. Pathogenesis of this disorder is essentially the result of mutations in the androgen receptor (*AR*) gene, but as clearly stated by Gottlieb et al. [4], “… solely identifying the genotype of an AIS individual may no longer be sufficient to draw conclusions about the effect of an *AR* mutation on the persons’ phenotype…”. The *AR* gene, located on chromosome Xq11–q12, contains eight exons and seven introns spanning ~90 kb of DNA. It encodes for the androgen receptor, which is a transcription factor and a member of the steroid hormone receptor family within the nuclear receptor superfamily [2]. *AR* is the only gene linked with AIS. More than 500 different *AR* mutations, distributed throughout the gene sequence, have been reported [3]. Furthermore, there is variable phenotypic expression related to specific mutations, with identical mutations linked to different phenotypes [3]. The clinical manifestations of AIS vary from the complete form (CAIS), where testosterone has no effect on sexual development and the genitals are entirely female, to milder degrees of undervirilization (the partial form; PAIS) or the mild form (MAIS) in which males have gynecomastia and/or infertility. The lifetime risk of development of neoplasms in patients with AIS, even though not defined accurately due to the small size of the analyzed series, was reported to be 0.8% for CAIS and 5.5%–15% for PAIS, with tumors originating from testicular germ cells, testicular stromal cells, or other mesenchymal cells [5–8].

To date, no cases of AIS with a documented history of colorectal cancer (CRC) have been described. In this paper, we describe the clinical and molecular analyses of a proband affected by PAIS who developed multiple CRCs. The patient presented with primary CRC at an early age (27 years). With the aim of identifying the constitutional genetic events that could have played a role in the early onset of CRC in the proband, we used a combination of two advanced high-throughput technologies: (i) high-resolution single nucleotide polymorphism (SNP) array analysis to investigate copy number variation (CNV) and uniparental disomy (UPD) events and (ii) whole exome sequencing (WES) analysis to investigate single nucleotide variants (SNVs) and small insertions and deletions (INDELs). A set of inherited germline genetic variations that may be responsible for CRC pathogenesis were identified in this patient.

## Case report

**Fig. 1:**
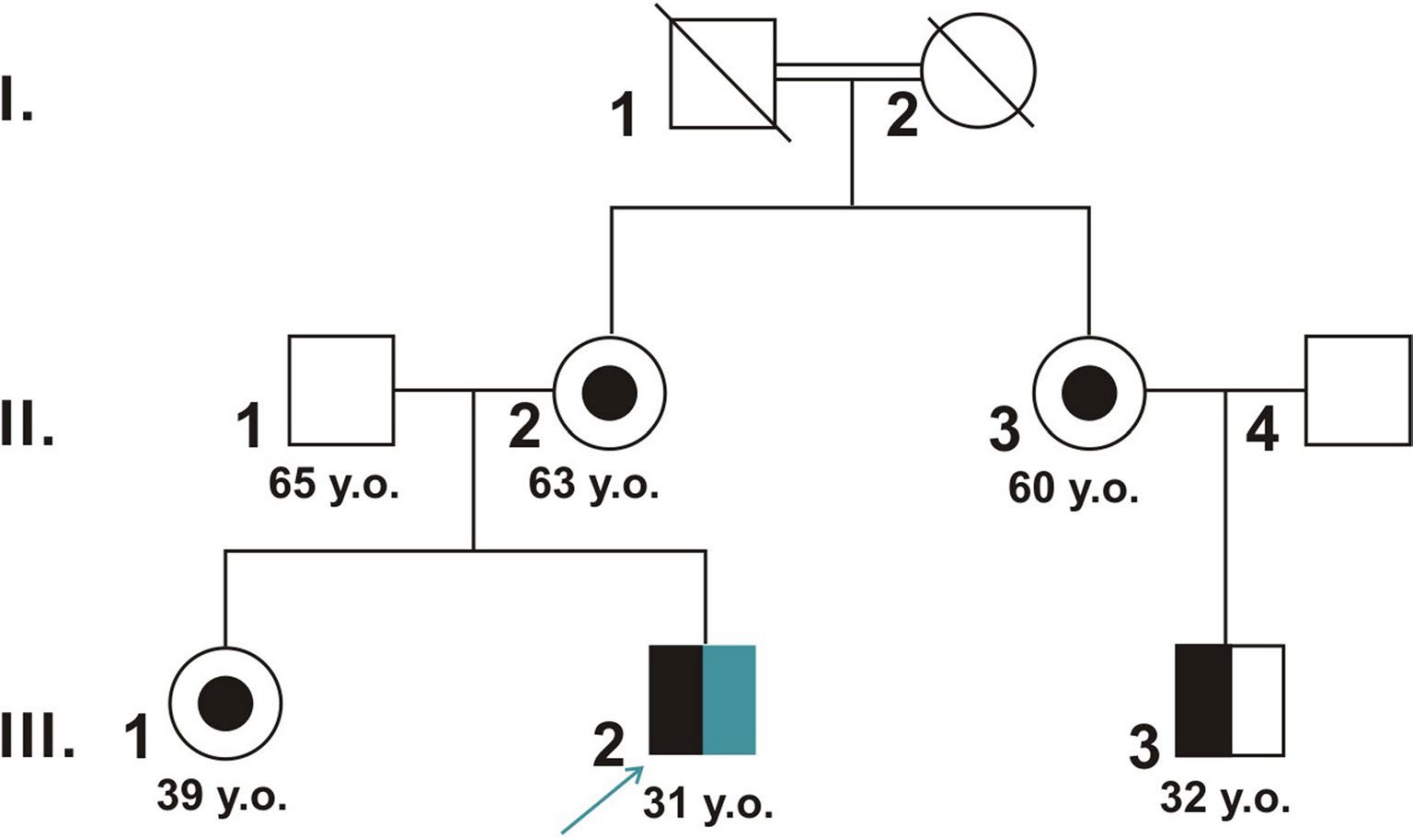
Pedigree of the family. *Squares* indicate males, and *circles* represent females. *Slashed symbols* indicate deceased individuals. *Unfilled symbols* indicate unaffected individuals. *Black dotted circles* indicate unaffected individuals carrying the androgen receptor (*AR*) gene mutation. *Half-black shaded symbols* represent individuals carrying the *AR* gene mutation who are affected by partial androgen insensitivity syndrome (PAIS). The *blue arrow* indicates the proband. The *half-blue shaded* symbol indicates that the proband presented with colorectal cancer (CRC). *y.o.* indicates the age at the time of the analysis. The proband developed primary CRC at 27 years old, metachronous CRC at 30 years old, and recurrent CRC at 31 years old

Herein, we report a 46,XY male patient (III.2 of the family presents in Fig. 1) who, at birth (1983), presented with undescended testes, severe hypospadias, and a micropenis and who received a clinical and molecular diagnosis of PAIS. At 6 months of age, adrenal hormone dosage results were normal, which excluded adrenal disease. Testosterone enanthate (250 mg every 2 weeks through intramuscular injection) was provided regularly from age 3 to 27 years. At 10 years of age, adrenocorticotropic hormone and beta human chorionic gonadotropin tests excluded a deficit in 5α-reductase. At 13 years of age, gynecomastia was recorded. The proband developed CRC at 27 years old and underwent a left hemicolectomy; at postoperative pathologic examination, the tumor was diagnosed as a G2, stage pT1N0 rectosigmoid adenocarcinoma. When he was 30 years old, a metachronous CRC was diagnosed and treated with segmental resection; at postoperative pathologic examination, the tumor was diagnosed as a G2, stage pT3N0 adenocarcinoma. The tumor relapsed locally 4 months later and was treated with radiotherapy, chemotherapy (capecitabine), and surgical proctectomy. At present, the disease is in progression.

The family history of CRC was negative. The proband was tested for germline mutations in the genes responsible for major CRC syndromes using Sanger sequencing [9–11], but no pathogenic mutations were identified (Additional file 1: Table S1). Immunohistochemical characterization of mismatch repair proteins as well as microsatellite instability analysis were performed as reported [12–14] on the primary CRC tissue. The normal staining pattern for all proteins is nuclear, and a case is considered positive only in the presence of nuclear staining of neoplastic cells. A case is considered defective for the expression of one protein only when there is a complete absence of nuclear staining of neoplastic cells in the presence of an unquestionable internal positive control represented by normal epithelial cells, stromal cells, muscle cells, or lymphocytes. The proband’s CRC tissue revealed no alterations in mismatch repair proteins.

The proband’s cousin (III.3) was also diagnosed with PAIS. He underwent gynecomastia, hypospadia, and cryptorchidism corrective surgeries, and testosterone enanthate (250 mg every 2 weeks through intramuscular injection) was provided in an irregular regimen from age 15 to 20 years. At present, the cousin is healthy.

Molecular analysis of the *AR* gene through Sanger sequencing [15] of DNA from both III.2 and III.3 revealed a missense mutation in *AR* exon 7 (NM_000044: c.G2522A; NP_000035.2: p.R841H). By segregation analysis, the *AR* mutation was found to be inherited from their healthy mothers (II.2, II.3), who were born from consanguineous parents. Furthermore, this variant was present in the DNA of the proband’s sister (III.1).

### Molecular analyses

Genomic DNA was extracted from whole blood specimens of the proband and his relatives using a QIAamp DNA Blood Maxi Kit (Qiagen, Hilden, Germany) and from secondary CRC and matched normal colon tissue of the proband using a QIAamp DNA mini Kit (Qiagen). Genomic DNA from whole blood samples from family subjects (II.1, II.2, II.3, III.2, and III.3) and from CRC tissue of the proband was subjected to high-resolution genome-wide SNP array analysis using a Cytoscan HD array (Affymetrix, Inc., Santa Clara, CA, USA) and WES using the SOLiD 5500xl WF platform (Life Technologies, Gaithersburg, MD, USA). WES was also performed on genomic DNA isolated from the normal colon mucosa of the proband. Two predictive tools, i.e., sorting intolerant from tolerant (SIFT) [16] and polymorphism phenotyping version 2 (PolyPhen-2) [17], were used to predict the effect of coding non-synonymous variants on protein function. To ensure the selection of genes associated with the currently best-characterized hereditary CRC syndromes, a review of the CRC panel-based clinical genetic tests listed in the GeneTest database was performed using “colorectal cancer” and “colon cancer” as key words. The final list of genes was then used to query in the PubMed and Online Mendelian Inheritance in Man (OMIM) databases to verify the association between the genes and a CRC predisposition. Genes that were found to be significantly somatically mutated in CRC patients and reported in the TumorPortal website (http://www.tumorportal.org) were included. A final list of 57 genes was used for analysis of the WES data (Additional file 2: Table S2; Additional file 3: Tables S3).

A network investigation of the identified mutated genes and genes harbored in the aberrant chromosomal regions was performed using Ingenuity^®^ Pathway Analysis (IPA^®^, QIAGEN Redwood City, http://www.qiagen.com/ingenuity; Version 23814503—23 March 2015).

### Identification of genetic events involved in PAIS

To identify the potential involvement of further genomic imbalances in the etiology of PAIS, we first evaluated the CNVs identified in the two affected cousins. Shared chromosomal regions identified as being deleted or duplicated in both cousins were carefully evaluated to verify their potential role in the pathogenesis of AIS. This analysis did not reveal the presence of CNVs that might have a role in the manifestation of the PAIS phenotype. Then, we scanned the exome data of the affected cousins using a list of 37 genes known to be responsible for DSD (Additional file 4: Table S4). This analysis confirmed the presence of the missense c.2522G > A mutation of the *AR* gene and excluded the occurrence of other causative genetic events being responsible for PAIS in both cousins (Additional file 5: Table S5).

### Identification of CNVs involved in the early onset of CRC

High-density SNP array (blood) analysis of the proband and his relatives was performed to investigate CNVs and UPD. Overall, in the proband, we identified 14 CNVs (Table 1; Additional file 6: Table S6): 4 (two losses and two gains) in gene desert chromosomal regions and 10 (two losses and eight gains) in chromosomal regions involving genes. All identified CNVs were also detected in the SNP array data of the CRC tissue. By comparison with the Database of Genomic Variants, 10 of the identified CNVs were found, with a frequency of <1% in healthy human control individuals from worldwide populations. Among the CNVs, four involved cancer-related genes, and one was a microRNA gene. Specifically, the identified copy number gains in the chromosomal regions 1p36.22, involving the PRY domain-containing SOCS box protein 1 (*SPSB1*) gene; 14q32.33, involving the immunoglobulin heavy constant gamma 1 (*IGHG1*) gene; Xp221.33 or Yp11.32, encompassing the cytokine receptor-like factor 2 (*CRLF2*) gene; and the copy number loss of chromosomal region 10q21.3, involving the intronic region of the catenin α 3 (*CTNNA3*) gene, have been previously identified in human cancers other than colon cancer. A microduplication of chromosome 22q11.22, encompassing *MIR650* gene, previously related to CRC, was also detected.

**Table 1:** List of chromosomal rearrangements detected in the proband through single nucleotide polymorphism array analysis

Chromosomal region	CN state	Known genes present in the indicated chromosomal region	III.2 Proband’s CRC tissue	II.1 Father’s blood sample	II.2 Mother’s blood sample	II.3 Aunt’s blood sample	III.3 Cousin’s blood sample
1p36.22^a^	3	***SPSB1***	✓		✓		
1q44	1	*OR2T29, OR2T34, OR2T10, OR2T11*	✓	✓			
4q13.2^a^	3	–	✓	✓	✓	✓	
5q15	1	–	✓		✓		
5q35.3^a^	3	*BTNL8, BTNL3*	✓				
8q24.23	1	–	✓	✓			
10q21.3^a^	1	***CTNNA3***	✓		✓		
12p13.2^a^	3	*PRH1*-*PRR4, TAS2R43*	✓		✓		
14q32.33^a^	3	*MIR8071*-*2, MIR8071*-*1, ELK2AP,* ***IGHG1*** *, FLJ00385, IGHD, MIR4507, MIR4538, MIR4537, MIR4539, KIAA0125, ADAM6, LINC00226*	✓	✓	✓	✓	✓
14q32.33^a^	3	–	✓				✓
17q21.31	3	*KANSL1, KANSL1*-*AS1*	✓	✓	✓		
22q11.22^a^	3	***MIR650*** *, MIR5571, IGLL5*	✓	✓	✓	✓	✓
Xp22.33^a^	3	***CRLF2***	✓	✓			
Yp11.32^a^							
Xq22.2^a^	2	*H2BFWT, H2BFM*	✓		✓		

Genes highlighted in bold have been shown to be relevant to cancer development

CN, copy number (State 1 indicates copy number loss; State 2 or 3 indicates copy number gain); *CRC* colorectal cancer; *SPSB1*, splA/ryanodine receptor domain and SOCS box-containing 1; *OR2T29*, olfactory receptor family 2 subfamily T member 29; *OR2T*, olfactory receptor family 2 subfamily T members; *BTNL8*, butyrophilin like 8; *BTNL3*, butyrophilin like 3; *CTNNA3*, cathenin alpha 3; *PRH1*-*PRR4*, PRH1-PRR4 readthrough; *TAS2R43*, taste 2 receptor member 43; MIR8071-2; MIR8071-1; *ELK2AP*, ELK2A, member of ETS oncogene family, pseudogene; *IGHG1*, immunoglobulin heavy constant gamma 1; *FLJ00385/IGHM*, immunoglobulin heavy constant mu; *IGHD*, immunoglobulin heavy constant delta; *KIAA0125*; MIR4507; MIR4538; MIR4537; MIR4539; *ADAM6*, ADAM metallopeptidase domain 6 (pseudogene); *LINC00226*, long intergenic non-protein coding RNA 22; *KANSL1*, KAT8 regulatory NSL complex subunit 1; *KANSL1*-*AS1*, KANSL1 antisense RNA 1; MIR650; MIR5571; *IGLL5*, immunoglobulin lambda like polypeptide 5; *CRLF2*, cytokine receptor-like factor 2; *H2BFWT*, H2B histone family member W testis specific; *H2BFM*, H2B histone family member M; *–*, no known genes are found

^a^The identified copy number variation (CNV) is reported in the database of genomic variants (DGV, http://www.dgv.tcag.ca/dgv/app/home) with a frequency <1%

No UPD events spanning whole chromosomes or chromosomal segments were detected in the proband. The segregation analysis of the 14 CNVs revealed that only one was de novo, whereas four were identified in both parents, three were inherited paternally, and five were inherited maternally.

### Identification of gene alterations involved in the early onset of CRC

WES generated an average of 456 × 10^6^ reads per sample, mapped to the reference genome with an average of 64.6% at a mean depth of 179-fold exon coverage (Table 2). The average depth coverage above 150, obtained for the proband, father, and cousin, far exceeded the normal standards applied for the identification of germline variants, making the results very robust. Furthermore, in the proband, the germline variants were cross validated in different tissues. Finally, the genotype of each proband variant could be further confirmed from the exome data of his parents.

**Table 2:** Metrics of whole-exome sequencing (WES) analysis of the specimens from the proband and his relatives entering into the present study

Metric	III.2 Proband’s blood sample	III.2 Proband’s normal colon tissue	III.2 Proband’s CRC tissue	II.1 Father’s blood sample	II.2 Mother’s blood sample	II.3 Aunt’s blood sample	III.3 Cousin’s blood sample
Total reads	801,798,464	631,026,099	631,377,353	633,336,574	98,744,957	98,744,957	397,006,863
Mapped reads	481,096,544	350,445,297	434,605,056	330,283,413	66,488,953	73,450,351	243,481,688
On-target reads	266,671,188	185,116,968	217,278,894	237,886,825	47,482,595	65,141,598	142,790,642
% mapped reads	60.0	55.5	68.8	52.2	26.8	74.4	61.3
% on-target reads	55.4	52.8	50.0	72.0	71.4	88.7	58.7
% covered target reads	96.7	92.6	91.6	94.9	86.7	88.6	94.9
Average depth coverage	288.6	200.3	235.1	257.4	51.4	70.5	154.5

**Fig. 2:**
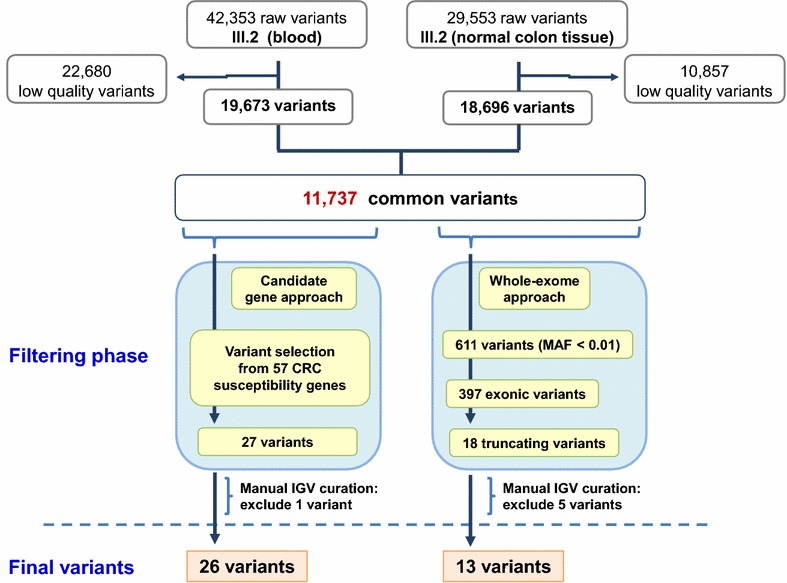
Analysis of the workflow applied to prioritize variants found in the whole-exome sequencing (WES) data from the proband. Two different sources of the proband’s DNA (blood and normal colon tissues) were analyzed with WES. A quality filter was first applied to minimize false positive variants, permitting us to obtain 19,673 and 18,696 variants in DNA in the blood and normal colon tissues from the proband, respectively. Since massively parallel sequencing can generate artifacts, a total of 11,737 variants shared between the two sources of DNA were used to apply a twofold strategy to identify variants associated with CRC. The first approach (“candidate gene approach”) selected 26 variants from the 57 CRC susceptibility genes; the second approach (“whole-exome approach”) selected 13 truncating variants with a minor allele frequency (MAF) <0.01. *IGV* the Integrative Genomic Viewer software

To identify variants associated with CRC in the proband, we carried out WES on DNA from blood and normal colon mucosa samples, and the variants shared between the two sources were analyzed using a candidate gene approach and a whole-exome approach (Fig. 2). The final set of prioritized variants was evaluated using the exome data from the relatives of the proband.

#### Candidate gene approach

By searching variants in the 57 prior candidate CRC-related genes without filtering criteria, we identified 27 variants present in the normal genomic DNA of the proband. After visually examining the variants using Integrative Genomic Viewer (IGV) software, one variant was excluded, thus reducing the final list to 26 variants (13 heterozygous and 13 homozygous) harbored in 17 CRC-related genes (Table 3; Additional file 6: Table S6).

**Table 3:** List of variants identified in the WES data of the proband (blood and normal tissue) through the “Candidate gene approach”

Gene symbol	Gene name	Mutation type	III.2 Proband’s blood sample	III.2 Proband’s normal colon tissue	III.2 Proband’s CRC tissue	II.1 Father’s blood sample	II.2 Mother’s blood sample	II.3 Aunt’s blood sample	III.3 Cousin’s blood sample	Inheritance
*APC*	Adenomatous polyposis coli	Synonymous	1/1	1/1	1/1	0/1	0/1	1/1	1/1	Both parents
			0/1	0/1	0/1	0/0	^a^	0/1	1/1	NV
			0/1	0/1	0/1	0/0	1/1	1/1	1/1	Mother
*AXIN2*	Axin 2	Intronic	1/1	1/1	1/1	1/1	0/1	1/1	1/1	Both parents
*BCOR*	BCL6 corepressor	Synonymous	1/1	1/1	1/1	1/1	1/1	1/1	1/1	Both parents
*CASP8*	Caspase 8, apoptosis-related cysteine peptidase	Missense	0/1	0/1	0/1	1/1	0/0	0/1	1/1	Father
*ELF3*	E74-like factor 3 (ets domain transcription factor, epithelial-specific)	Synonymous	0/1	0/1	0/1	0/1	^a^	0/0	0/1	Father
*EPHB2*	EPH receptor B2	Intronic	0/1	1/1	1/1	0/1	0/1	1/1	0/1	Both parents
		Synonymous	1/1	1/1	1/1	1/1	^a^	^a^	1/1	NV
*ERBB2*	Erb-b2 receptor tyrosine kinase 2	Missense	1/1	1/1	1/1	1/1	0/0	0/1	0/1	De novo
*GOT1*	Glutamic-oxaloacetic transaminase 1, soluble	Synonymous	0/1	0/1	0/1	0/1	0/0	0/0	0/0	Father
*KRAS*	Kirsten rat sarcoma viral oncogene homolog	3′-UTR	1/1	1/1	1/1	1/1	^a^	1/1	1/1	NV
			1/1	1/1	1/1	1/1	1/1	0/1	0/1	Both parents
		Synonymous	1/1	1/1	1/1	1/1	^a^	1/1	1/1	NV
*MLH1*	mutL homolog 1	Missense	1/1	1/1	1/1	0/1	0/0	0/0	0/1	De novo
*MSH6*	mutS homolog 6	Synonymous	0/1	0/1	0/1	0/0	0/1	0/1	0/1	Mother
*NR4A2*	Nuclear receptor subfamily 4, group A, member 2	3′-UTR	1/1	1/1	1/1	1/1	0/1	1/1	1/1	Both parents
*NTN4*	Netrin 4	Missense	0/1	0/1	0/1	1/1	0/0	0/1	0/1	Father
*PMS2*	PMS2 postmeiotic segregation increased 2 (*S. cerevisiae*)	Intronic	1/1	1/1	1/1	0/1	1/1	0/0	0/1	Both parents
*POLE*	Polymerase (DNA directed), epsilon, catalytic subunit	Intronic	0/1	0/1	0/1	0/1	0/0	0/0	0/0	Father
		Synonymous	0/1	0/1	0/1	1/1	0/1	0/1	0/1	Both parents
			0/1	0/1	0/1	0/0	^a^	^a^	0/0	NV
			0/1	0/1	0/0	1/1	0/0	0/0	0/0	Father
*PTPRJ*	Protein tyrosine phosphatase, receptor type, J	Intronic	1/1	0/1	0/1	1/1	0/1	^a^	0/1	Both parents
		Missense	1/1	1/1	1/1	0/1	1/1	0/1	0/1	Both parents
*SMAD4*	SMAD family member 4	Intronic	0/1	0/1	0/1	0/1	0/1	0/0	1/1	Both parents

“0” refers to reference allele; “1” refers to alternative allele; 0/1 indicates heterozygous variant; 1/1 indicates homozygous variant

*UTR* untranslated region

^a^Targeted region presenting low coverage (<10X)

Analysis using the 1000 genomes database revealed that 24 of the 26 variants had a minor allele frequency (MAF) >5%, indicating that these SNVs could be considered common variants, whereas the remaining two identified in the polymerase (DNA directed), epsilon, catalytic subunit (*POLE)* (chr12:133233977G > A, synonymous mutation) and protein tyrosine phosphatase, receptor type, J (*PTPRJ*) (rs11382316, intronic mutation) genes represented rare variants.

We focused our analysis on variants that produced an altered protein product and found that five of the 26 variants were non-synonymous. Among these, only the rs1058808 polymorphism of the erb-b2 receptor tyrosine kinase 2 (*ERBB2*) gene was predicted to be deleterious by both SIFT (damaging, tolerance index score ≤0.05, low confidence prediction) and PolyPhen-2 (possibly damaging, tolerance index score in the 0.453–0.956 range) (Additional file 7: Table S7).

A search for the identified variants in the MAF file reporting somatic variations of the 233 samples from the Cancer Genome Atlas (TCGA) project indicated that they were not described in the CRC data set. The Human Gene Mutation Database (HGMD) [18], Leiden Open Variation Database (LOVD) [19], and Human Genome Variation Society (HGVS) and ClinVar databases [20], which were used to assess the pathogenicity of these variants, indicated no evident pathogenicity associated with CRC.

The variants identified in the proband were then evaluated in the WES data of his relatives. Among the 13 heterozygous variants, six were inherited paternally, two were inherited maternally, and three were present in both parents; for the remaining two variants, due to low coverage of the targeted analyzed regions, the inheritance was not assessable. Among the 13 homozygous variants, eight were inherited from both parents. Two of the homozygous variants included one allele that was transmitted from the father, while presumably the SNV harbored in the other allele occurred *de novo*. The inheritance of three of the homozygous variants was not assessable. A majority of the identified variants (22 of 26) was also detected in the proband’s cousin.

#### Whole-exome approach

We focused our search on variants functionally annotated and predicted to truncate the protein products, i.e., frameshift insertions and deletions and non-sense mutations. Variants were excluded if they were present in the 1000 Genomes Project and dbSNP138 databases at only MAF >0.01. Among 11,737 variants shared by the two sources of normal DNA of the proband, 18 truncating variants were identified, but the manual revision of these through IGV software subsequently excluded five of them. The remaining 13 variants were in a heterozygous state (Table 4) and were essentially confirmed by the WES data of the proband’s relatives, i.e., four SNVs were inherited maternally and three were inherited paternally, whereas the presence of the other three in the aunt and cousin suggested possible inheritance from the proband’s mother. One rare variant in the *HYDIN* gene was identified in a heterozygous state in all members of the family.

**Table 4:** List of truncating mutations identified in the proband and his relatives

Gene symbol	Gene name	Mutation type	III.2 Proband’s blood sample	III.2 Proband’s normal colon tissue	III.2 Proband’s CRC tissue	II.1 Father’s blood sample	II.2 Mother’s blood sample	II.3 Aunt’s blood sample	III.3 Cousin’s blood sample	Inheritance
*ADHFE1*	Alcohol dehydrogenase, iron containing, 1	*Stop gain*	0/1	0/1	0/1	0/0	^a^	0/1	0/1	NV
*AHNAK2*	AHNAK nucleoprotein 2	*Stop gain*	0/1	0/1	0/1	0/0	^a^	0/1	0/1	NV
*BTN3A3*	Butyrophilin, subfamily 3, member A3	*Stop gain, intronic*	0/1	0/1	0/1	^a^	0/0	0/0	0/0	NV
*CHMP2B*	Charged multivesicular body protein 2B	*Stop gain, intronic*	0/1	0/1	0/1	0/1	0/0	0/0	0/0	Father
*DENND2D*	DENN/MADD domain containing 2D	*Stop gain, intronic*	0/1	0/1	0/1	0/0	0/1	0/1	0/1	Mother
*HYDIN*	HYDIN, axonemal central pair apparatus protein	*Frameshift del*	0/1	0/1	0/1	0/1	0/1	0/1	0/1	Father/mother
*KALRN*	Kalirin, RhoGEF kinase	*Stop gain, intronic*	0/1	0/1	0/1	0/0	0/1	0/1	0/1	Mother
*GPR114*	ADGRG5, adhesion G protein-coupled receptor G5	*Frameshift del*	0/1	0/1	0/1	0/0	^a^	0/1	0/0	NV
*MEP1A*	Meprin A, alpha (PABA peptide hydrolase)	*Stop gain*	0/1	0/1	0/1	0/0	0/1	^a^	0/1	Mother
*SCCPDH*	Saccharopine dehydrogenase (putative)	*Frameshift ins*	0/1	0/1	0/1	^a^	^a^	0/0	0/0	NV
*TMEM177*	Transmembrane protein 177	*Stop gain*	0/1	0/1	0/1	0/1	0/0	0/0	0/0	Father
*TGM4*	Transglutaminase 4	*Stop gain*	0/1	0/1	0/1	0/1	0/0	0/0	0/0	Father
*ZNF844*	Zinc finger protein 844	*Frameshift del*	0/1	0/1	0/1	0/0	0/1	0/1	0/1	Mother

“0” refers to reference allele; “1” refers to alternative allele; 0/1 indicates heterozygous variant; 1/1 indicates homozygous variant

^a^Targeted region presenting low coverage (<10X)

### Network and pathway analysis

To test putative enrichment for canonical pathways, disease, biological functions, and molecular networks, the genes residing in CNVs and those harboring truncating mutations were analyzed using QIAGEN’s Ingenuity^®^ Pathway Analysis.

**Fig. 3:**
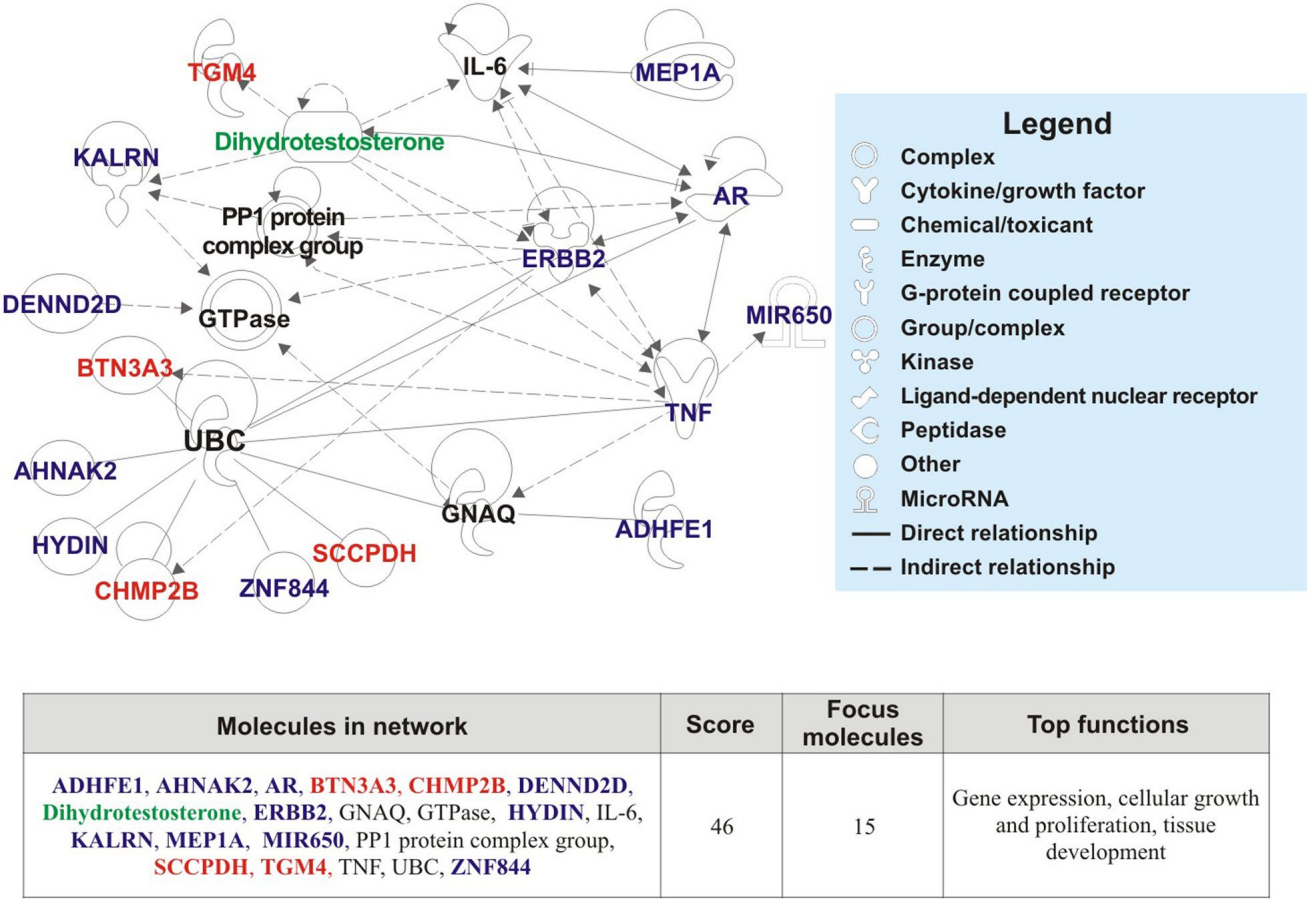
Network of genes and molecules inferred from Ingenuity® Pathway Analysis (IPA). The 13 genes exhibiting truncating variants were combined with the erb-b2 receptor tyrosine kinase 2 (*ERBB2*) gene harboring the rs1058808 single nucleotide polymorphism (SNP), which was predicted to be deleterious by the sorting intolerant from tolerant (SIFT) and polymorphism phenotyping version 2 (PolyPhen-2) tools, the microRNA-650 (*MIR650*) gene (a colorectal cancer-related gene), and the androgen receptor (*AR*) gene harboring the pathogenic rs9332969 SNP to test the putative enrichment of the canonical pathways, disease and biological functions, and molecular networks. When the short list of altered genes was examined using the IPA software, a single pathway (top functions: gene expression, cellular growth and proliferation, tissue development) with a high score (46, *P* < 0.001) was identified. Genes highlighted in *blue* were identified to be altered in III.2 (proband) and III.3 (cousin). Genes highlighted in *red* were found to be altered only in subject III.2. The dihydrotestosterone molecule is highlighted in *green*

Significant gene association was observed only when the gene list was reduced to *AR* (mutated and causative of PAIS in the proband), *MIR650* (already related to CRC pathogenesis and present in a rearranged chromosomal region), *ERBB2* (determined to be functionally impaired by the candidate gene approach), and the 13 genes harboring truncating variants (identified by the whole-exome approach). In fact, 14 (87.5%) of these 16 genes were found to be connected to each other in a unique relevant network called “Gene expression, cellular growth and proliferation, tissue development” (IPA score 46) (Fig. 3). When a testosterone metabolite (dihydrotestosterone) was added to the network, indirect interactions with genes of the network, including *ERBB2*, were found in addition to the expected direct interaction with AR. Table 5 summarizes the main characteristics of the altered genes related to this network and their genetic association with the analyzed samples.

**Table 5:** Summary of the characteristics of genes connected in the “Gene expression, cellular growth and proliferation, tissue development” network

Gene symbol	Gene name	Exonic variant type	Accession number	Amino acid change	Inheritance
PAIS					
*AR*	Androgen receptor	Missense	NM_000044	p.R841H	Mother
Candidate gene approach					
*ERBB2*	Erb-b2 receptor tyrosine kinase 2	Missense	NM_001005862	p.P1140A	Father/de novo
Whole-exome approach					
*ADHFE1*	Alcohol dehydrogenase, iron containing, 1	Stop gain	NM_144650	p.R6X	NV
*AHNAK2*	AHNAK nucleoprotein 2	Stop gain	NM_138420	p.W4640X	NV
*BTN3A3*	Butyrophilin, subfamily 3, member A3	Stop gain	NM_006994	p.Trp171X	NV
*CHMP2B*	Charged multivesicular body protein 2B	Stop gain	NM_014043	p.Arg22X	Father
*DENND2D*	DENN/MADD domain containing 2D	Stop gain	NM_024901	p.Arg16X	Mother
*HYDIN*	HYDIN, axonemal central pair apparatus protein	Frameshift del	NM_001270974	p.I3904fs	Father/mother
*KALRN*	Kalirin, RhoGEF kinase	Stop gain	NM_007064	p.Arg10X	Mother
*MEP1A*	Meprin A, alpha (PABA peptide hydrolase)	Stop gain	NM_005588	p.Q296X	Mother
*SCCPDH*	Saccharopine dehydrogenase (putative)	Frameshift ins	NM_016002	p.V55fs	NV
*TGM4*	Transglutaminase 4	Stop gain	NM_003241	p.W269X	Father
*ZNF844*	Zinc finger protein 844	Frameshift del	NM_001136501	p.L346fs	Mother

Gene content	Chromosomal aberration	Inheritance
SNP array		
*MIR650, MIR5571, IGLL5*	22q11.22 (23,004,886–23,258,369) × 3	Father/mother

## Discussion

This study provided a deep genomic analysis using SNP array and WES data of the constitutive DNA of a patient harboring the missense c.2225G > A (p.R841H or rs9332969) mutation of the *AR* gene. It has been reported that the R841H protein variation influences its binding to and interactions with androgens [21]; however, this substitution does not result in complete *AR* functional disruption [22] and gives rise to variable expression of the AIS phenotype [2, 23], suggesting that alterations in other genes may modulate and affect the final phenotype. Our SNP array and WES analysis of both affected cousins in this study did not reveal the presence of additional causative genetic events responsible for the PAIS phenotype, thus strongly supporting the pathogenic role of the p.R841H variant in PAIS.

Our proband patient developed multiple CRCs at an early age, suggesting a hallmark of inherited cancer susceptibility along with familial aggregation [24, 25]. Moreover, it has been recently reported that genetic instability could explain the early onset of CRC [18]. Germline alterations in the major CRC-associated genes adenomatous polyposis coli (*APC*), mutY homolog (*MUTYH*), mutL homolog 1 (*MLH1*), mutS homolog 2 (*MSH2*), and mutS homolog 6 (*MSH6*) were previously excluded. In this study, we employed two advanced high-throughput technologies, namely SNP array and WES, to investigate the involvement of other genetic susceptibility mechanisms responsible for early onset of CRC.

Among the 14 CNVs identified by the SNP array, four involved genes (*SPSB1*, *CTNNA3*, *IGHG1*, and *CRFL2*) have been implicated in tumorigenesis, however, other than in colon cancer, thus only one rearrangement, a duplication of the chromosomal region 22q11.22, may be of interest and relevant to this study. The amplified chromosomal region included *MIR650*, which has among its targets the promoter region of the NDRG family member 2 (*NDRG2*) tumor suppressor gene where it has been reported to be able to repress *NDRG2* gene expression at the transcriptional level in CRC [26].

Among the 26 variants identified by WES following the candidate gene approach, a literature search revealed that the *APC*, caspase 8, apoptosis-related cysteine peptidase (CASP8), *ERBB2*, *MLH1*, and *PTPRJ* gene variants have been previously investigated to verify their roles in cancer predisposition (see all the references in Additional file 7: Table S7). Even if no clear pathogenic mutations responsible for CRC insurgence were identified, it is worth noting that rs1058808 in the *ERBB2* gene, present in the heterozygous state, is harbored in the regulatory C-terminal domain of the ErbB2 protein. This protein represents a hub of the protein–protein interaction network associated with the ErbB2 receptor, which plays a crucial role in signaling cascades regulating cell growth, differentiation, and migration [27]. Furthermore, previous studies investigating the role of the rs1058808 polymorphism have suggested that it may represent a functional polymorphism inducing the overexpression of the ErbB2 protein [28, 29]. At present, in a series of 104 sporadic CRC patients, three mutations in the *ERBB2* kinase domain have been found [30]; however, the mutation identified in the proband patient was located in a different region. Finally, our WES approach identified 13 genes harboring loss-of-function variants in the heterozygous state in the proband.

The analysis of the prioritized genes harboring the loss-of-function variants combined with the *ERBB2*, *AR*, and *MIR650* genes through IPA software revealed that 14 of the 16 genes were connected in a unique network, called the “Gene expression, cellular growth and proliferation, tissue development” network. The core molecular hub in this network is represented by seven genes (*MIR650*, alcohol dehydrogenase, iron containing 1 [*ADHFE1*], charged multivesicular body protein 2A [*CHMP2B*], DENN domain containing 2D [*DENND2D*], kalirin RhoGEF kinase [*KALRN*], meprin A subunit alpha [*MEP1A*], and transglutaminase 4 [*TGM4*]) connected with *AR* and *ERBB2*, revealing an altered cross-talk between these two signaling pathways. From the literature, four of these six genes (*CHMP2B*, *DENND2D*, *KALRN*, and *TGM4*) have been previously identified as being involved in oncogenesis, while 3 (*MIR650*, *ADHFE1*, and *MEP1A*) may be related to CRC development and differentiation (see all the references in Additional file 8: Table S8). Considering the genes connected in the network but that were not altered in sequence, the presence of the interleukin-6 (*IL*-*6*) gene and its relation to *MEP1A* are also noteworthy. Indeed, IL-6 has been shown to be increased in Meprin KO mice [31], and IL-6 has been found to be up-regulated in colon cancer tissues and significantly associated with an increased risk of relapse [32]. Moreover, it should be noted that, among these 14 connected genes, five were not altered in the cousin. Testosterone, indirectly connected with *TGM4* and *ERBB2*, was provided continuously for 24 years to the proband, including developmental years, whereas only 5 years of discontinuous treatment was provided to the cousin after age 15 years.

In physiologic conditions and in the presence of an active *AR*, the activity of testosterone and dihydrotestosterone hormones can be modulated by (i) a classic genomic model mediated by the cytosolic AR protein and (ii) a non-genomic model mediated by the functional membrane AR [33]. Since membrane AR activation via testosterone induces cytosolic signaling cascades involving molecules belonging to the *ERBB2* pathway, it is tempting to hypothesize the possible existence of cross-talk between testosterone, membrane AR, and ErbB2 protein [34–38]. Considering the role of testosterone in CRC development, the literature indicates that the risk of CRC shows sexual dimorphism, with a higher incidence and earlier onset in men [39]. Whether this disparity depends on protective factors in women, tumor-promoting factors in males, or both, is unknown. A recent epidemiologic study indicated that, in men with functional AR protein, higher levels of testosterone and sex hormone-binding globulin were associated with a decreased risk for developing CRC, but lower androgenicity may increase the risk of developing CRC in males [40, 41]. A tumor-promoting effect of testosterone, likely not dependent on AR activity, has been reported via the elegant use of the APC^Pirc/+^ (Pirc) rat model, suggesting that sex disparity in the development of colonic adenomas depends directly on the effect of testosterone, rather than on a protective effect of female hormones [42]. Testosterone has been shown to stimulate the growth of lung and colon cancer cells in vitro, a mechanism that can be halted with anti-androgens [43, 44]. A population-based study that aimed to verify the association between plasma testosterone and overall cancer risk revealed that increased levels of plasma testosterone were associated with an increased risk of early death after cancer, but not with risk of cancer incidence [45]. Putting all these factors together, these observations suggest a potential effect of testosterone on cancer development and prognosis.

Thus, we speculate that testosterone treatment may have played a role in the initiation of cancer in the colon tissue of the proband, probably exerting a direct interaction with the poorly or non-functional cytosolic AR protein or an indirect action through the membrane AR protein. This could have led to the activation of a cytosolic signaling cascade involving the ErbB2 protein, which may have a functional effect in the patient.

## Conclusions

The etiology of CRC is complex and multifactorial, involving an interplay between multiple genetic and environmental factors. Our in-depth genetic analysis of the proband and his family supports the hypothesis that the identified genetic alterations, in a multi-hit model of the inheritance of common and rare variants in multiple genes, might have contributed to the early onset of CRC in combination with environmental factors, such as a potential iatrogenic effect due to a continuous high-dose treatment with testosterone during developmental years. To confirm the relevance of the identified network of genes and molecules in CRC predisposition, further AIS families and preclinical model systems should be analyzed. Moreover, these findings highlight the importance of these types of studies to develop a better understanding of the molecular basis of a disease and, consequently, for better clinical management of patients.

## Consent

All experiments were approved by the Ethical Committee of Fondazione IRCCS Istituto Nazionale dei Tumori (Milan, Italy). A written informed consent was obtained from all subjects for publication of individual patient data.

## Additional files

10.1186/s40880-016-0115-1 Variants of tumor genes detected by Sanger sequencing in the proband.
10.1186/s40880-016-0115-1 List of genes associated with inherited predisposition to colorectal cancer (CRC).
10.1186/s40880-016-0115-1 List of genes significantly mutated in 233 colorectal tumors (TCGA project).
10.1186/s40880-016-0115-1 List of disorder of sex development (DSD) genes used to filter exome sequencing variants.
10.1186/s40880-016-0115-1 List of single nucleotide variants (SNVs) and small insertions and deletions (INDELs) detected in WES data of III.2 and III.3 subjects.
10.1186/s40880-016-0115-1 Chromosomal rearrangements of cancer function-related genes detected in the proband through single nucleotide polymorphism.
10.1186/s40880-016-0115-1 List of variants identified in whole-genome sequencing (WES) data of the proband (blood sample and normal colon tissue) through the candidate gene approach.
10.1186/s40880-016-0115-1 List of truncating mutations identified in the proband and his relatives.
